# P-1045. Decreasing Frequency of Invasive Aspergillus due to *Aspergillus terreus* Coinciding with the Introduction of Anti-Mold Azole Prophylaxis: A 30 Year Study of Invasive Aspergillosis at a Tertiary Cancer Center

**DOI:** 10.1093/ofid/ofae631.1235

**Published:** 2025-01-29

**Authors:** Hiba Dagher, Ray Y Hachem, Andrea Haddad, Ying Jiang, Dimitrios P Kontoyiannis, Jishna Shrestha, Robin Sherchan, Peter Lamie, Jennifer Makhoul, Patrick Chaftari, Anne-Marie Chaftari, Issam I Raad

**Affiliations:** UT MD Anderson Cancer Center, Houston, Texas; MD Anderson UT, Houston, Texas; MD Anderson Cancer Center, Houston, Texas; The University of Texas MD Anderson Cancer Center, Houston, Texas; The University of Texas MD Anderson Cancer Center, Houston, Texas; MD Anderson Cancer Center/ Baylor College of Medicine, Houston, Texas; MD Anderson Cancer Center / Baylor College of Medicine, Houston, Texas; UT MD Anderson Cancer Center, Houston, Texas; University of Texas Health Science Center at Houston/MD Anderson Cancer Center, Houston, TX; UT MDAnderson Cancer Center, Houston, TX; MD Anderson UT, Houston, Texas; MD Anderson UT, Houston, Texas

## Abstract

**Background:**

Invasive aspergillosis (IA) is a significant cause of morbidity and mortality in patients with haematological malignancy (HM) patients and hematopoietic stem cell transplant (HSCT) recipients. *A. terreus* is well known to be more resistant to amphotericin B regimens and is associated with worse outcomes as compared to *non-terreus Aspergillus* species. After the first introduction of effective anti-mold azole in May 24,2002, there has been limited data showing the change in in etiology of culture documented IA. We retrospectively reviewed all HM and HSCT patients with a positive culture of Aspergillosis who were treated for either definite or probable IA over the past three decades (1993-2023).

**Methods:**

We compared baseline characteristics, antifungal prophylaxis and treatment between patients infected with *A. terreus* and those infected with *non-terreus Aspergillus* between July 1993 and July 2023 with a follow up period of 12 weeks. We examined the rate of *A. terreus* across the 3 decades 1993 – 2003, 2004 – 2013, and 2014-2023.

**Results:**

A total of 699 patients with culture -documented IA were analyzed, 537 with *A. non-terreus* species and 162 with *A. terreus*. Type of underlying malignancy (HM and HSCT), neutropenia as well as GVHD were similar in both groups. Anti-mold prophylaxis was similar in both groups. After the introduction of anti-mold azoles, the rate of *A. terreus* decreased significantly from 35.9 % between 1993-2003 to 11.2% between 2004-2013 (p< 0.0001) and from 35.9% between 1993-2003 to 16.7% between 2014-2023 (p< 0.0001). ICU stay and mechanical ventilation were more common among patients with *A. terreus* (p=0.002). All-cause mortality at 6 weeks and at 12 weeks was significantly higher in patients with *A. terreus* (p< 0.01). Similarly, IA-associated 6 weeks mortality was significantly higher among patients with *A. terreus* (43 % vs 29%, p< 0.01).

**Conclusion:**

The rate of IA due to *A. terreus* decreased significantly with the introduction of anti-mold azoles. However, IA caused by *A. terreus* continues to have poor prognosis in HM patients.

**Disclosures:**

**Dimitrios P. Kontoyiannis, MD**, AbbVie: Advisor/Consultant|Astellas Pharma: Advisor/Consultant|Astellas Pharma: Grant/Research Support|Astellas Pharma: Honoraria|Cidara Therapeutics: Advisor/Consultant|Gilead Sciences: Advisor/Consultant|Gilead Sciences: Grant/Research Support|Gilead Sciences: Honoraria|Knight: Advisor/Consultant|Merck: Advisor/Consultant|Scynexis: Advisor/Consultant

